# Insights into the formation of CdSe nanoplatelets using a flow reactor†

**DOI:** 10.1039/d4nr03804e

**Published:** 2024-10-29

**Authors:** Julia Irmhild Marie Funk, Benedikt Sochor, Sarathlal Koyiloth Vayalil, Horst Weller

**Affiliations:** a Department of Physical Chemistry, University of Hamburg Grindelallee 117 20146 Hamburg Germany julia.funk@uni-hamburg.de; b Deutsches Elektronen-Synchrotron DESY Notkestrasse 85 22607 Hamburg Germany; c Applied Science Cluster, University of Petroleum and Energy Studies UPES Dehradun 248007 India

## Abstract

In recent years, the anisotropic semiconductor nanoplatelets (NPLs) gained interest due to their unique optical properties, which depend primarily on their thickness. However, the formation mechanism behind the zinc blende CdSe NPLs remains unclear. Several theories were presented and discussed, but a concrete mechanism has not yet been found with evidence. Here, we want to present a synthesis of CdSe NPLs in a flow reactor with a liquid precursor, enabling *in situ* absorbance measurements. The flow reactor allows for more control in obtaining early-stage synthesis samples, which were *ex situ* examined with optical spectroscopy, transmission electron microscopy, as well as small-angle and powder X-ray diffraction. Our results show that CdSe magic size clusters (MSCs) formed prior to the formation of CdSe NPLs, indicating that these CdSe MSCs are necessary for the initial CdSe NPLs growth.

## Abstract 2

A novel synthesis to produce CdSe nanoplatelets in a flow reactor is introduced. Furthermore, the connection between CdSe magic-size clusters and the growth of these anisotropic nanoplatelets is investigated.

## Graphical abstract

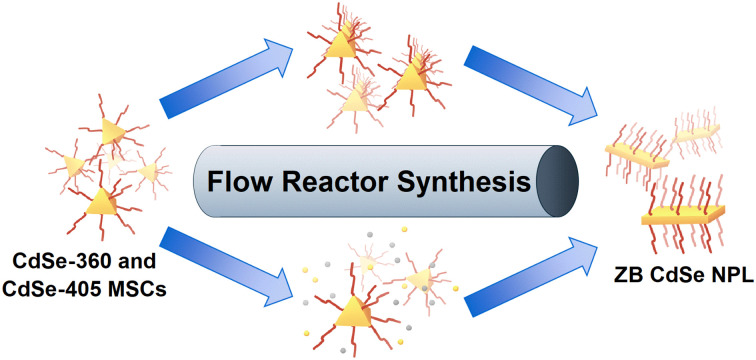

Recently, the synthesis of anisotropic semiconductor nanoplatelets (NPLs) has been introduced and investigated.^1–5^ Compared to semiconductor CdSe quantum dots, CdSe NPLs of the same material exhibit much narrower absorption and emission peaks.^1,2^ These narrow peaks arise from the strong confinement of the exciton through the thickness of the CdSe NPLs.^3^ This strong confinement results in the emergence of a light-hole and a heavy-hole transition in the absorption of the CdSe NPLs.^2,3^ As a consequence of the exciton confinement, only a Stokes shift of a few nanometers is visible in the emission.^1,3^ One exciting aspect is that the thickness of the CdSe NPLs and, thus, their unique optical properties can be controlled precisely through the synthesis conditions.^3,4^ Through adding halides during the synthesis, like cadmium chloride, thicker CdSe NPLs can be obtained.^5^

Higher temperatures and longer reaction times also lead to thicker CdSe NPLs.^6^ CdSe NPLs can also be modified by growing either a shell or a crown structure onto them, thus enabling type-I, quasi-type-II, and type-II heterostructures.^7–9^ Moreover, these semiconductor nanoplatelets have the potential to be used as low-lasing threshold gain media.^10^ While many ways exist to produce and modify CdSe NPLs, their formation mechanism is still not fully understood. Some theories try to explain how these anisotropic zinc blende CdSe NPLs form.^6,11,12^ One theory explains the growth of CdSe NPL based on an intrinsic instability in growth kinetics.^6,12^ The other theory is based on an oriented attachment growth mechanism with an intraparticle ripening process.^11^ A few groups also tried to apply these theories with other materials of the same anisotropic shape.^13,14^ However, no definite explanation as been found to date. Studying the reaction *in situ* is desired to get further insights into the formation mechanism.^15^ Furthermore, controlling the reaction parameters like time and temperature is crucial. One method for achieving better control of the synthesis conditions would be the usage of a flow reactor.^15,16^ Most CdSe NPLs syntheses rely on one solid educt; hence, they are unsuited for a flow reactor.^12,17,18^

Here, we want to present a new CdSe NPLs synthesis approach, using only liquid educts to study the formation of the CdSe NPLs in a flow reactor *in situ*. We successfully obtained 3 monolayer (ML) and 4 ML thick CdSe NPLs through the flow reactor. The synthesis absorbance is tracked *in situ*, providing valuable real-time insights. Further analysis of these samples is done through *ex situ* optical measurements, small-angle X-ray scattering (SAXS), and powder X-ray diffraction (powder XRD). We observe and identify CdSe Magic-Size Clusters (MSCs) in the early synthesis stages before forming the anisotropic CdSe NPLs. Our results indicate that these CdSe MSCs are necessary for forming CdSe NPLs and, thus, are a crucial part of a formation model of CdSe NPLs.

To prevent reactor clogging, we use a CdSe NPLs synthesis with a pure liquid precursor (see ESI† for further details). For the selenium component in the synthesis, pure selenium was dissolved in 1-octadecene (ODE). The cadmium components consist of cadmium oleate in ODE, to which acetic acid was added. With these components, we managed to find a synthesis that can easily be scaled up to allow mass production of the CdSe NPL. We also used an already established flow reactor system for this work.^15^ All presented results are based on precursors with the same precursor composition.

Fig. 1a shows absorbance spectra measured *in situ* at 210 °C with various reaction times, as well as the unreacted precursor in a 10 cm flow reactor. It should be noted that after the reaction took place in the oven at the given reaction times, the reaction was quenched in a heat exchanger before the *in situ* measurements in the flow reactor system (see ESI† for more information about the flow reactor setup). Starting at 55 s, a small peak is forming at 360 nm. This peak increases until a reaction time of 65 s is set for the flow reactor. If the reaction time is increased to 70 s, the same peak visibly decreases in intensity. The decrease of this peak continues until the final reaction time of 85 s. Based on the peak's position at 360 nm, it is likely that CdSe MSCs have formed.^19,20^

**Fig. 1:**
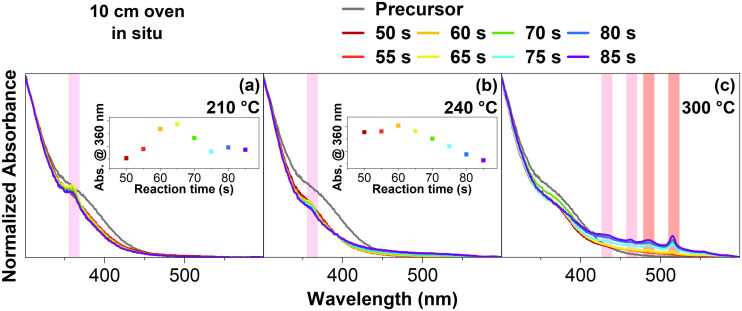
*In situ* absorbance spectra of reactor samples in a 10 cm oven at different temperatures and reaction times. (a) Shows the reaction conducted at 210 °C, (b) at 240 °C, and (c) at 300 °C. At 210 °C CdSe MSCs are clearly visible (light pink), at 300 °C the formation of CdSe NPL is apparent (pink and dark pink).

As it is difficult to determine the exact structure and composition of MSCs,^21^ they are usually assigned based on their absorption maximum.^22^ Consequently, the found CdSe MSCs in Fig. 1a can be assigned to CdSe-360. If we attempt to assign a shape to these MSCs, they would probably have a tetrahedral shape.^23^ This is based on the synthesis conditions, specifically the used carboxylates with oleic acid and acetic acid. Found by other groups, carboxylates usually favor non-stochiometric MSC shapes.^24–26^

Fig. 1b now shows various reaction times at 240 °C. Similar to before, a peak at 360 nm forms at 60 s. However, the peak decreases in intensity, starting at a reaction time of 65 s. Furthermore, the intensity of all reaction times is much lower than that of the unreacted precursor. It becomes apparent that the same CdSe-360 MSCs as before have formed but with a much lower intensity, indicating that the MSCs may undergo a different process at higher temperatures of 240 °C.

Fig. 1c shows the reaction conducted at 300 °C. At 50 s, tiny peaks at 435 nm, 463 nm, 481 nm, and 513 nm start to form. These distinct peaks at 435 nm and 462 nm correspond to the light- and heavy-hole transitions of 3 ML CdSe NPLs.^27^ The other two peaks at 481 nm and 513 nm correspond to 4 ML CdSe NPLs.^27^ These four peaks increase in their intensity until 85 s. Based on the steeper slope around the CdSe NPLs peaks, some CdSe dots have also formed.^15^ Furthermore, it should be noted that we have a laminar flow profile in the relatively short 10 cm flow reactor oven (see ESI†). These flow conditions probably impact why NPLs have formed at such high temperatures. These *in situ* absorption spectra indicate that CdSe-360 MSCs are forming before CdSe NPLs are formed.

To receive further clarification about these MSCs, photoluminescence excitation (PLE) spectra are shown in Fig. 2a. These samples are synthesized in a 90 cm flow reactor oven. Starting at 200 °C and 20 s, a shoulder at 405 nm is forming. It is important to note that this present feature does not correspond to 2 ML CdSe NPLs. For 2 ML CdSe NPLs, a double peak at 372 nm and 393 nm would be expected.^28^ Unlike the previous clear peak at 360 nm, there is an apparent shoulder at 405 nm, which may correspond to CdSe-405 MSCs. This may indicate that there is another second CdSe MSCs population in the synthesis. Based on previous reports, multiple CdSe MSC sizes are commonly observed at the same time.^22,29^ The same shoulder shows a slight blue-shift once the reaction time increases to 40 s at 200 °C. This blue-shift continues until 220 °C and 60 s. At 230 °C and 60 s, a small peak at 435 nm becomes visible, corresponding to 3 ML CdSe NPL.^17^ Following at 240 °C and 60 s, the same peak increases in intensity while the shoulder from before is not undergoing a blue-shift. Instead, at 240 °C and 120 s, the shoulder red-shifts. These excitation spectra strongly suggest that the CdSe MSCs become another intermediate before the formation of CdSe NPLs. Based on the blue-shift, the intermediate needs to decrease their size. In the oriented attachment theory by Peng *et al.*, the CdSe seeds they used also had to undergo an intraparticle-size ripening to form two-dimensional embryos.^11^ In our case, the MSCs formed are likely to undergo a similar process before forming CdSe NPLs. These intermediates would also explain the observed blue shift in the excitation spectra. Once these intermediate stages reach a specific thickness and a sufficient lateral size, the typical peaks for the CdSe NPLs appear, similar to our observed excitation spectra. This can be explained by the three-dimensional confinement of the CdSe MSCs, which is different from the one-dimensional confinement of CdSe NPLs, which arises from their thickness. Their lateral sizes are so large that there is no confinement of the exciton in these two directions within a CdSe NPL.

**Fig. 2:**
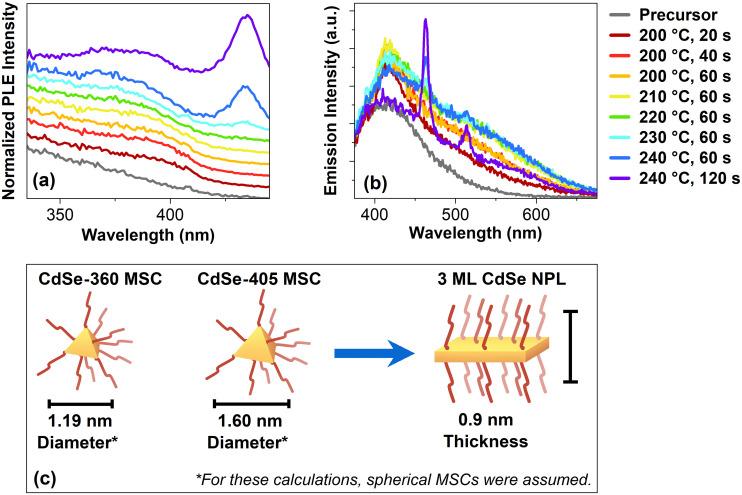
(a) Excitation spectra of the CdSe NPLs flow reactor samples from a 90 cm oven. Here, the emission was set to 463 nm. (b) Emission spectra of the CdSe NPLs flow reactor samples, excited at 350 nm. (c) Schematic drawing of a CdSe-360 MSC, CdSe-460 MSC and a 3 ML CdSe NPL. The diameter was determined using the method developed by Yu *et al.*^30^

Fig. 2b shows the emission of the reactor samples with an excitation wavelength of 350 nm. Compared to the unreacted precursor, the intensity at 418 nm increases for the earliest sample at 200 °C and 20 s. With an extended reaction time of 40 s, the intensity increases, especially around 500 nm. The intensity increases for the following samples until 210 °C and 60 s when the maximum is reached. Compared to the sample before at 200 °C and 20 s, the emission for this sample is broad and showcases a plateau-like structure around 530 nm. In the following sample, at 220 °C and 60 s, the intensity decreased. Also, once the temperature rises to 230 °C, a small peak at 463 nm appears, corresponding to 3 ML CdSe NPLs.^17^ At 240 °C and 60 s, another peak at 514 nm occurs, now corresponding to 4 ML CdSe NPLs.^5^ Similar to before, their intensity increases when the reaction time extends to 120 s. Interestingly, the emission plateau around 530 nm decreases when the NPLs form. In context to the previous discussion, the emission plateau may originate from the intermediates forming between the MSCs and the NPLs.

Assuming tetrahedral CdSe MSCs, the structural difference between the found CdSe-360 MSCs, CdSe-405 MSCs, and the CdSe NPLs is also depicted in Fig. 2c. The exact structure and composition of the two CdSe MSCs are unknown. To determine the diameter for these CdSe MSCs, we assume a spherical shape and calculate the size according to Yu *et al.*^30^ According to these calculations, the CdSe-360 MSCs may have a diameter of 1.19 nm, and the CdSe-405 MSCs of 1.60 nm. Because the MSCs in this work are non-spherical in their diameters, their actual diameters are larger than these calculated diameters. The 3 ML CdSe NPLs can have any size in their lateral dimension. However, their thickness is reported as 0.9 nm.^31^ Thus, if CdSe MSCs are forming before the formation of CdSe NPLs, they probably need to reduce their size in one dimension to 0.9 nm in order to form CdSe NPLs.

Fig. 3a shows the emission spectra of the samples from the 90 cm oven with an excitation wavelength of 435 nm. In the first samples, starting from 200 °C and 20 s up until 210 °C and 60 s, no peaks are visible. Once the reaction reaches 220 °C and 60 s, a peak appears at 464 nm. This peak belongs to the 3 ML CdSe NPLs.^17^ The intensity of this peak increases with higher reaction temperatures and longer reaction times. Furthermore, another sharp peak at 514 nm occurs for the samples starting at 230 °C and 60 s. This peak belongs to the 4 ML CdSe NPLs.^5,17^ Based on the results, the 3 ML CdSe NPLs formed first, and only with an increase in reaction time we observed 4 ML CdSe NPLs. These spectra agree with the current literature on CdSe NPLs formation.^6^ Increasing the temperature or reaction time will increase the thickness of the CdSe NPLs.^6^ Transmission electron microscopy (TEM) images in Fig. 3b and c show the unpurified product we received at the end of the flow reactor synthesis.

**Fig. 3:**
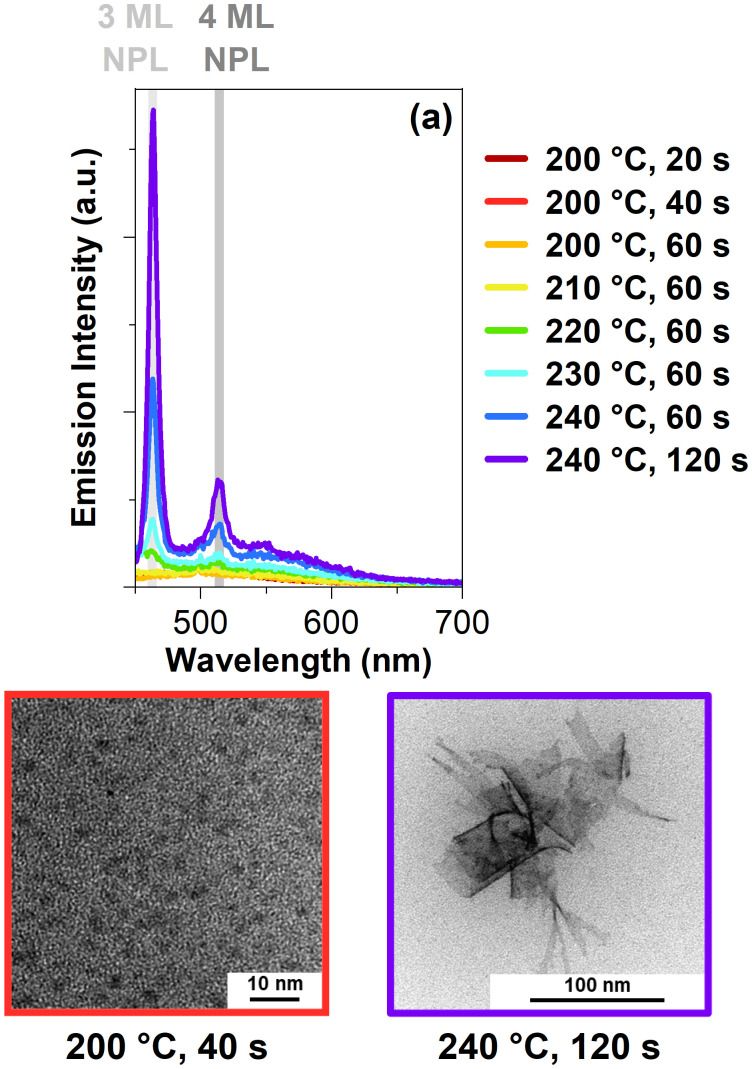
(a) Emission spectra of the CdSe NPLs flow reactor samples measured with a laser excitation of 435 nm. The emission position of 3 ML CdSe NPLs is marked in light grey, and the position for 4 ML CdSe NPLs in dark grey. (b) and (c) show TEM images of CdSe MSCs and CdSe NPLs in the unpurified flow reactor samples.

CdSe “seeds” can be observed with a size of 2.1 ± 0.4 nm at 200 °C and 40 s, which is in the size regime of CdSe MSCs.^21,32^ Compared to the calculated values above, the CdSe MSCs sizes from the TEM are slightly bigger. As the raw, unpurified product was evaluated which includes the solvent and ligands, was evaluated, HRTEM images could not be measured. Consequently, slight variations between the calculated values and the values found in the TEM images are expected based on the resolution limit. In Fig. 3c, a TEM image is presented of the two-dimensional CdSe NPLs in the raw product. Their length is 25 ± 19 nm, and width is 75 ± 26 nm, resulting in an area size with an approximate value of 2066 ± 2110 nm. As depicted in Fig. 3c, the lateral sizes of these CdSe NPL vary a lot which may arise from the one-pot synthesis approach for the flow reactor synthesis. Some of these CdSe NPLs, shown in Fig. 3c, roll up because of their large size, which results in an approximate lateral size value.

For further information on the formation of the CdSe NPLs using a flow reactor synthesis method, we have performed *ex situ* small-angle X-ray scattering (SAXS) measurements at the beamline P03 of PETRA III (DESY Hamburg, Germany).

Fig. 4a shows the first SAXS patterns, including fits in Fig. 4b, to gain information on the particle's shape and size (see ESI† for the measurements and the fitting models). As the exact MSC structure is unknown, for the following fits, a spherical shape of the CdSe MSCs and intermediates was assumed except for the final sample, which used a parallelepiped fit that can be applied for CdSe NPLs. Firstly, it becomes evident that the low q region reveals spherical-like particles for the sample at 200 °C and 20 s, which gradually turn into anisotropic platelets for the final sample at 240 °C and 120 s (Fig. 4a). In the initial stages of the synthesis, the samples at 200 °C with a reaction of 20 s and 60 s contain MSCs based on the fitting results in Fig. 4b. The first sample at 200 °C and 20 s yielded CdSe MSCs with a diameter of 1.8 nm, which once again aligns with the size regime of MSCs.^21,32^ Another sphere fit was applied on the following sample at 200 °C and 60 s, obtaining a diameter of 3.2 nm. This diameter increase can be explained as the diameter is only an average value. As the MSC structure is not spherical and only has this assumed shape for the fits, the increased radius indicates an agglomeration depending on their orientation. This agglomeration may indicate the beginning of the formation of an intermediate structure, as suggested in the previous results. In our measurements, we have no hints for an oriented attachment process, as we would expect the formation of a superlattice.^33^ However, the sample 210 °C, 60 s and the following samples 220 °C, 60 s and 230 °C, 60 s indicate agglomeration in their low q region. Moreover, the following fits indicate a decrease in size (sample 210 °C, 60 s and sample 220 °C, 60 s) before the as-formed intermediate's size increases, starting with the sample 230 °C, 60 s. Based on the previous results, the intermediate's size decrease could explain the blue-shift observed in the excitation spectra, as a smaller structure would cause a stronger exciton confinement. Furthermore, the sample at 230 °C, 60 s not only grows in size, based on the SAXS fits, but also exhibits the 3 ML CdSe NPL light-hole peak at 435 nm.^27^ This change indicates that the as-formed intermediate turned into a CdSe NPL. The following fits indicate further growth for the sample at 240 °C and 60 s. Finally, the CdSe NPL with a thickness of 0.9 nm could be obtained for the sample at 240 °C and 120 s. This obtained thickness also fits the size of 3 ML CdSe NPLs in the literature.^34^ Moreover, the fit indicates CdSe NPLs sizes of 13.8 nm × 161.3 nm which suit the TEM image of the CdSe NPLs above. However, the structure of the intermediate remains unknown. Before the CdSe NPL structure is obtained, one possible explanation of the intermediate structure is an egg-tray-like structure, as proposed for the formation of PbS sheets.^13^ We also performed powder X-ray diffraction (powder XRD) on our CdSe NPLs (see Fig. S7 in the ESI† for more information). Our CdSe NPLs were reported to have most likely a cubic zinc blende structure, as expected from the literature.^35^ In both samples, another peak at 20 degrees is visible, likely arising from remaining organic materials.^36^

**Fig. 4:**
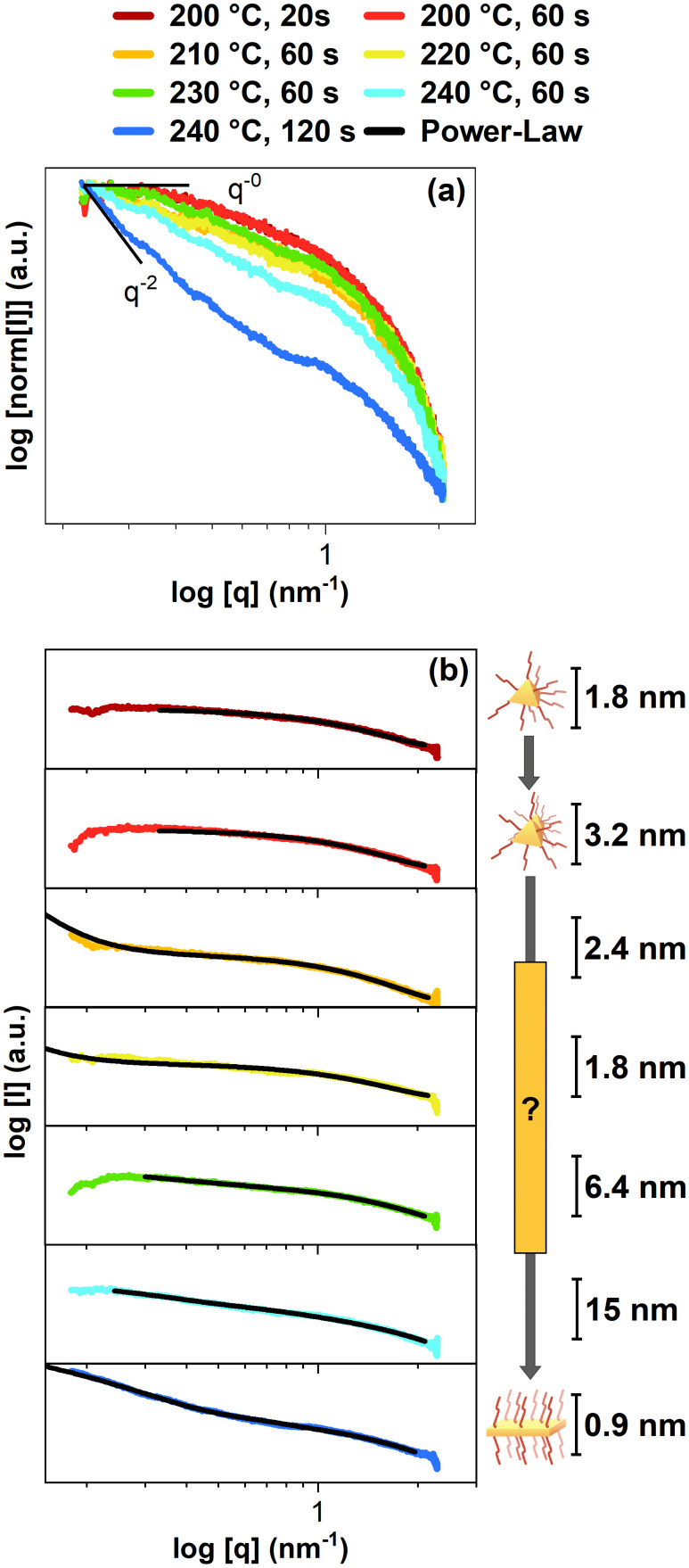
Normalized SAXS pattern (a) of the CdSe reactor samples at different temperatures and reaction times. (a) In these SAXS sequences, the change in power law from q-0 to q-2 indicates the transformation from CdSe MSCs to CdSe NPLs. (b) Fitted SAXS data reveal average solvation sizes of 1.8 nm for CdSe MSC, agglomerated CdSe MSCs, an unknown structure for an intermediate, and finally, CdSe NPLs with a thickness of 0.9 nm. Various fitting models were applied to obtain the fit for each individual SAXS pattern. More information on the fitting models are in the ESI.†

Based on the previous results, we propose a formation mechanism for the CdSe NPLs, as illustrated in Scheme 1.

**Scheme 1:**
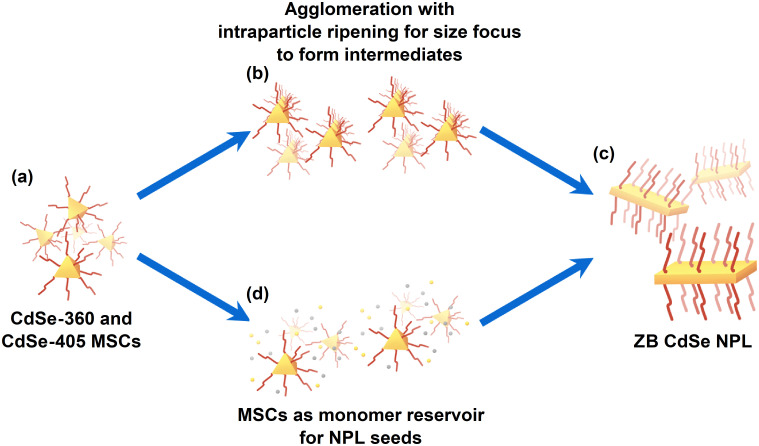
Possible formation mechanism for CdSe NPLs. (a) At the beginning, CdSe MSCs form. From there on, there are two options. (b) Shows the agglomeration pathway, which leads to the formation of an intermediate before the finished zinc blende CdSe NPLs form in (c). The other possible path is for the MSCs to act as monomer reservoir in (d) before turning to CdSe NPLs.

This proposed mechanism was developed using a novel synthesis approach with liquid precursors, which is different to the literature in which at least one solid is commonly used.^12,17,18^ Even if the physical state differs for the short carboxylate used in our synthesis approach, all the key components found in our synthesis approach are also present in the “typical” CdSe NPL synthesis methods, which use a solid. These key components to obtain these anisotropic CdSe NPLs are the short and long carboxylate, in our case, acetic acid and cadmium oleate, respectively. The other chemicals used in our flow reactor synthesis approach are the same as for the other “typical” CdSe NPL synthesis methods. As a result, our findings have a high probability of being applied to the general formation of CdSe NPLs in other “typical” CdSe NPL synthesis methods.

At first, CdSe MSCs form (Scheme 1a). From there on, two possible pathways are available. One possible pathway is the agglomeration with intraparticle ripening to form intermediates, as shown in Scheme 1b. Its mechanism leans towards the oriented attachment process proposed by Peng *et al.*^11^ They used spherical CdSe seeds with diameters between 1.7 to 2.2 nm, which riped to anisotropic CdSe NPLs, in their synthesis, alongside short and long carboxylates in stearic acid and cadmium acetate. In their theory, the long carboxylate is used to stabilize the dot-shaped nanocrystals and the polar {100} facet of the nanocrystals while also leading the short carboxylate to the reactive surfaces of the nanocrystals. The short carboxylate then converts the seed to the anisotropic NPL.^11^

Similar to their proposed formation mechanism, we observed MSCs to form with a larger diameter, which must undergo a size-focusing process to form thinner NPLs at the very end (Scheme 1c). As we also used short and long carboxylates like Peng *et al.*,^11^ it is likely that these carboxylates in our flow reactor synthesis fulfill similar roles to their proposed theory. However, we do not see a superlattice in our SAXS measurements, making an oriented attachment unlikely. In another group, the Petukhov group, the synthesis of CdSe NPLs was observed to exhibit the coexistence of both isotropic and anisotropic particles at the early stages of the process.^37^

Scheme 1d provides another potential pathway. In this scenario, the CdSe MSCs serve as a monomer reservoir for CdSe NPLs nuclei. This theoretical pathway would support the findings of Riedinger's group.^6^ They proposed that concentrated droplets in a solution promote an island-nucleation-limited growth.^6^ Hence, if the dissolution of CdSe MSCs increases the monomer concentration rapidly in a local part, there is a possibility that this acts like a droplet to promote the growth of the anisotropic CdSe NPLs.

Hence, both pathways may play a vital role in forming CdSe NPL. It is possible that CdSe MSCs agglomerate together and then undergo an intraparticle ripening to form an intermediate. This size focus can happen through a partial dissolution process. The intermediate can then act as a seed to growth in lateral dimensions by the attachment of the as-formed CdSe monomers from the dissolution process. Thus, the reality behind CdSe NPLs formation may be the combined interaction of both formation processes. Based on our results, it is evident that an intermediate is forming prior to the formation of the CdSe NPLs.

In summary, we performed a CdSe NPLs synthesis in a flow reactor to unravel the formation mechanism of CdSe with *in situ* and *ex situ* examinations of our early-stage synthesis samples. We report the formation of CdSe MSCs in the earliest stages of the CdSe NPLs synthesis based on their absorption of 360 nm, their emission of 418 nm, and the SAXS measurements.

With an increase in reaction temperature and time, we first observe a blue-shift in excitation and emission spectra before we observe 3 ML CdSe NPLs with absorption peaks of 435 nm and 463 nm and an emission peak of 464 nm. This indicates the formation of an intermediate structure prior to the formation of the CdSe NPLs, which is also supported by the emission spectra. TEM images also prove that CdSe MSCs and CdSe NPLs are synthesized in a flow reactor with the same precursor solution.

The SAXS measurements also showcase a transition from MSCs, to an intermediate before the formation to CdSe NPLs. Furthermore, a zinc blende structure was determined based on the powder XRD results. These CdSe MSCs are most likely necessary for forming the zinc blende CdSe NPLs. Our model demonstrates that these MSCs either undergo a size ripening process, including an agglomeration or dissolve to act as monomer reservoirs for NPLs formation. The knowledge we have obtained from our results thus provides significant information for understanding the mechanism of NPLs formation and growth. Additionally, the access to the liquid-synthesis in a flow reactor allows an easy scale-up, thus enabling production of large quantities of CdSe NPLs for various applications.

## Data availability

The data supporting this article have been included as part of the ESI.†

## Conflicts of interest

There are no conflicts to declare.

## Supplementary Material

NR-016-D4NR03804E-s001
