# Supplementary material for: Insights into the formation of CdSe nanoplatelets using a flow reactor
Source: Nanoscale. 2024 Oct 29;16(46):21309–16. doi: 10.1039/d4nr03804e (PMC11529821; doi:10.1039/d4nr03804e)
Supplement: NR-016-D4NR03804E-s001 [file NR-016-D4NR03804E-s001.pdf]

Supplementary Information (SI) for Nanoscale.
This journal is © The Royal Society of Chemistry 2024

INSIGHTS INTO THE FORMATION OF CDSE NANOPLATELETS USING A FLOW REACTOR

Julia Imrhild Marie Funk,*[a] Benedikt Sochor,[b] Sarathlal Koyiloth Vayalil,[b,c] and Horst Weller[a]

SUPPORTING INFORMATION

EXPERIMENTAL SECTION.

CHEMICALS. Acetic acid (AcOH; ≥99.5%) was purchased from Chem Solute. Cadmium oxide (CdO; ≥99.9%) was purchased from Merck. 1-Octadecene (ODE; ≥90%), oleic acid (OA; ≥90%), selenium powder (Se; ≥ 99.9%) were purchased from Sigma-Aldrich. Ethanol (EtOH; ≥99.8%) was purchased from VWR. *n*-Hexane (≥95%) was purchased from Fisher Chemicals. All chemicals were used without any further purification.

SELENIUM-ODE PRECURSOR (0.19 M) PREPARATION. In a 250 mL round-bottom flask, 150 mg of Se-powder and 100 mL ODE were degassed for 2 hours at 100 °C. The mixture was then heated and stirred under nitrogen flow for 8 hours at 240 °C until all the selenium solids dissolved. The yellow liquid was cooled down and then stored under nitrogen at room temperature.

REACTOR PRECURSOR PREPARATION. In a 250 mL round bottom flask, 190 mg of CdO, 1425 µL of OA, and 200 mL of ODE were heated for 1 hour at 240 °C under nitrogen flow. The brown suspension turned into a clear, colorless solution. This product was cooled down to 110 °C. Then, the mixture was degassed at 110 °C for 4 hours. The mixture was cooled down to room temperature. Under nitrogen flow, 5 mL of the 0.19 M Se-ODE solution was added.

The mixture was degassed again at room temperature for one hour. After degassing, the mixture was stored under nitrogen. Before the synthesis in the flow reactor, 55 µL AcOH was added to the mixture under nitrogen flow. Then, the mixture was stirred vigorously for at least 30 min before being used in the flow reactor.

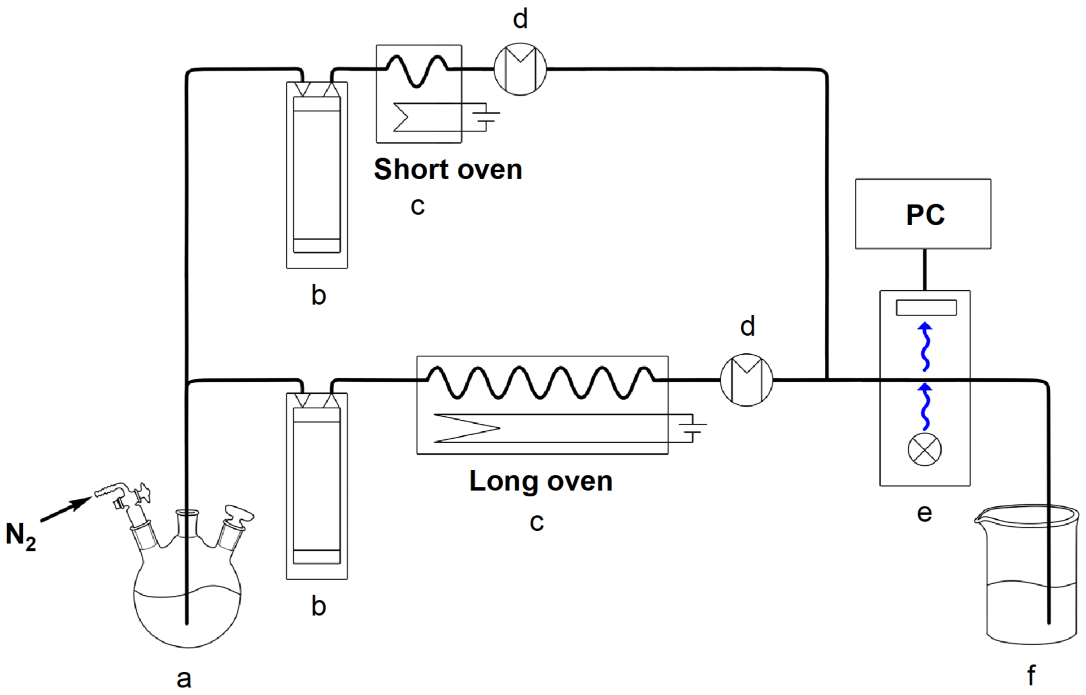

Scheme S1. Flow reactor setup. (a) The Reactor precursor solution is pumped into the NEMESIS syringe pumps (b). The reactor precursor solution is then heated in the ovens in (c) before the reaction is quenched to room temperature in the heat exchangers in (d). In situ spectroscopy is possible in the quartz glass flow cells in (e), which are connected to a PC. The reactor samples are then collected in (f) in a beaker or vials.

SYNTHESIS OF CDSE NPLs USING A FLOW REACTOR. Before the synthesis, the flow reactor system was first flooded with ODE. Then, the reactor precursor, purged with nitrogen, was pumped into the NEMESYS syringe pumps, and the flow reactor system was inundated with the reactor precursor. The flooding with ODE and the reactor precursor were measured using the built-in absorbance spectrometer and the MultiSpec 2 program to control the flooding of

the system with the correct solution. The oven's reaction temperature was set through the Elotech R2500 PID controller. The flow rate and, thus, the reaction time within the ovens were precisely controlled with the NEMESYS QMIX program. During the currently running experiment, samples were only taken after the system was flooded two times with the presently produced reaction product.

FLOW REACTOR SETUP. A previously owned setup was used with minor modifications, shown in scheme S1. The precursor solution in (a) is connected with 1/16 inches PEEK tubing to two individual NEMESYS syringe pumps from CETONI in (b). Each syringe pump had a volume of 25 mL and was used to control the flow rate. They also had built-in pressure sensors to observe the pressure during the reaction. The two syringes were connected to two titanium ovens in (c) with 1/16 inches of more PEEK tubes and additionally with tubes, ferrules, nuts, and unions out of titanium. Each oven had a different length of either 10 cm, 30 cm, or 5 m, depending on which oven was built into the line. Every oven can be heated individually with their heating cartridges. The heating cartridges are connected to an Elotech R2500 PID controller with 16 zones to monitor and control the temperature. With this construction, by varying the temperature and flow rates and the oven lengths, we gain precise control over the reaction times and temperatures of our precursor solution and the finished products. Behind every oven, a heat exchanger (d) is installed to cool the precursor solution directly at room temperature. After the heat exchangers, the tubes are combined through a T-piece and connected to an optical flow cell in (e). The optical flow cell by Knauer has a path length of 3 mm and quartz glass windows, thus enabling in situ absorbance measurements. Optical fibers connect the flow cell to the spectrometer. The reaction samples can be collected in (f) at the very end. The collected samples can then be further examined ex situ.

IN SITU OPTICAL SPECTROSCOPY. As described in the flow reactor setup, in situ absorbance measurements of the samples happen within the optical flow cell. The spectrometer is connected to a PC, the program MultiSpec 2 by Tec5 allows to track and save the in situ absorbance spectra. Before every measurement, reference spectra are taken by flooding the system with ODE. The samples are collected after one residue time within the flow reactor has passed.

CDSE NPL BATCH SYNTHESIS. The batch reaction is similar to the flow reactor precursor synthesis above. In a 50 mL round bottom three-neck flask, 38 mg of CdO, 285 µL of OA, and 40 mL of ODE were heated for 45 min at 240 °C under nitrogen flow. The colorless product was cooled down to 110 °C and was degassed for 1 hour at this temperature. After degassing, the colorless solution was cooled down to room temperature and 1 mL of the 0.19 M Se-ODE solution was added. The mixture was degassed again at room temperature for 30 minutes. Then, 11 µL AcOH was added to the mixture under nitrogen flow. The mixture was stirred vigorously for at least 30 min before heating up to 240 °C. At 240 °C, the reaction was kept there for 10 minutes before rapidly cooling it down to room temperature.

SAMPLE PREPARATION FOR EX SITU OPTICAL SPECTROSCOPY. The collected reactor samples were not purified further. All samples were diluted 1 out of 5 with *n*-hexane in a quartz glass cuvette with a path length of 10 mm. Absorbance measurements were recorded using a Varian Cary 50 UV/vis spectrometer. Here, the reference spectra used were pure *n*-hexane. Excitation and emission spectra were recorded using a Fluoromax 50 spectrometer. There, an excitation wavelength of 350 nm and 435 nm were used for the emission spectra.

The excitation spectra ranged from 325 to 445 nm, setting the emission monochromator to 463 nm.

SAMPLE PREPARATION FOR EX SITU TRANSMISSION ELECTRON MICROSCOPY. The collected reactor samples were not purified further. All samples were diluted 1 out of 50 with *n*-hexane. From these diluted samples, 10 µL were dropped onto carbon-coated copper grids.

SAMPLE PREPARATION FOR EX SITU SAXS MEASUREMENTS. The reactor samples were carefully filled into quartz glass capillaries without further purification. The capillaries were sealed using clay and glue to minimize solvent evaporation.

SYNCHROTRON SAXS EXPERIMENTS. All experiments were performed at the beamline P03 of PETRA III (DESY, Hamburg, Germany). For the experimental setup, the X-ray wavelength was fixed at 1.023 Å, and a Pilatus 2M detector (Dectris, Baden, Switzerland), with a pixel size of 172 µm, was used to collect the scattering patterns. The sample-to-detector distance was fixed at (3910 ± 2) mm. Due to the beam size of 30 × 30 μm^2, each capillary was scanned vertically over a range of 50 mm (step size: 0.1 mm, illumination time: 0.1 s per image). All collected images were summed up before data reduction. To obtain the scattering curves, the images were azimuthally averaged, and the resulting 1D intensity pattern was scaled to represent absolute intensities using the standard procedures from the literature.[1,2]

SAMPLE PREPARATION FOR EX SITU XRD MEASUREMENTS. The final reactor sample was purified. For the purification, 5 mL of the raw product, 5 mL *n*-hexane and 5 mL ethanol were centrifuged at 7000 g for 10 min. The supernatant was discarded and the step was repeated. Afterwards, the precipitate was collected in 2 mL *n*-hexane and stored under nitrogen in a glass vial. The purified sample was drop casted onto a Si-wafer. Materials Project archive was used to obtain reference crystal structure data for CdSe.[3]

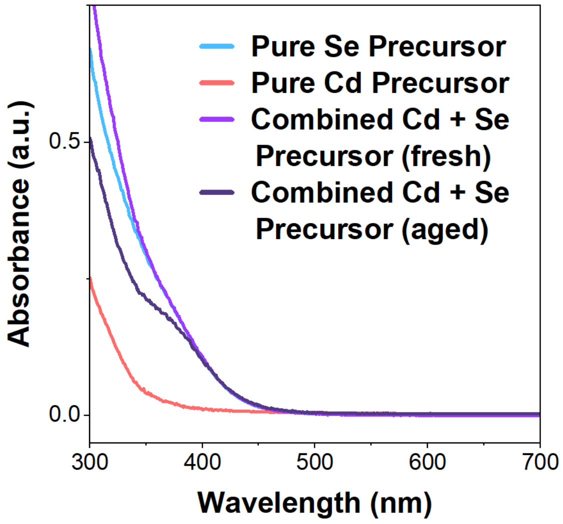

Figure S2. Ex situ absorbance spectra to determine the source of the absorbance of the finished reactor precursor, shown in two purple shades. The pure cadmium components in the precursor show a negligible absorbance in the range from 300 to 350 nm. Compared to the pure cadmium precursor, the pure selenium precursor has an absorbance until ~ 430 nm is visible. This selenium precursor is used for the baseline-corrected absorbance. Another experiment was performed with the finished reactor precursor, consisting of both the cadmium and the selenium components. A shoulder structure is visible when the precursor is aged (dark purple), resembling the MSCs measurements.

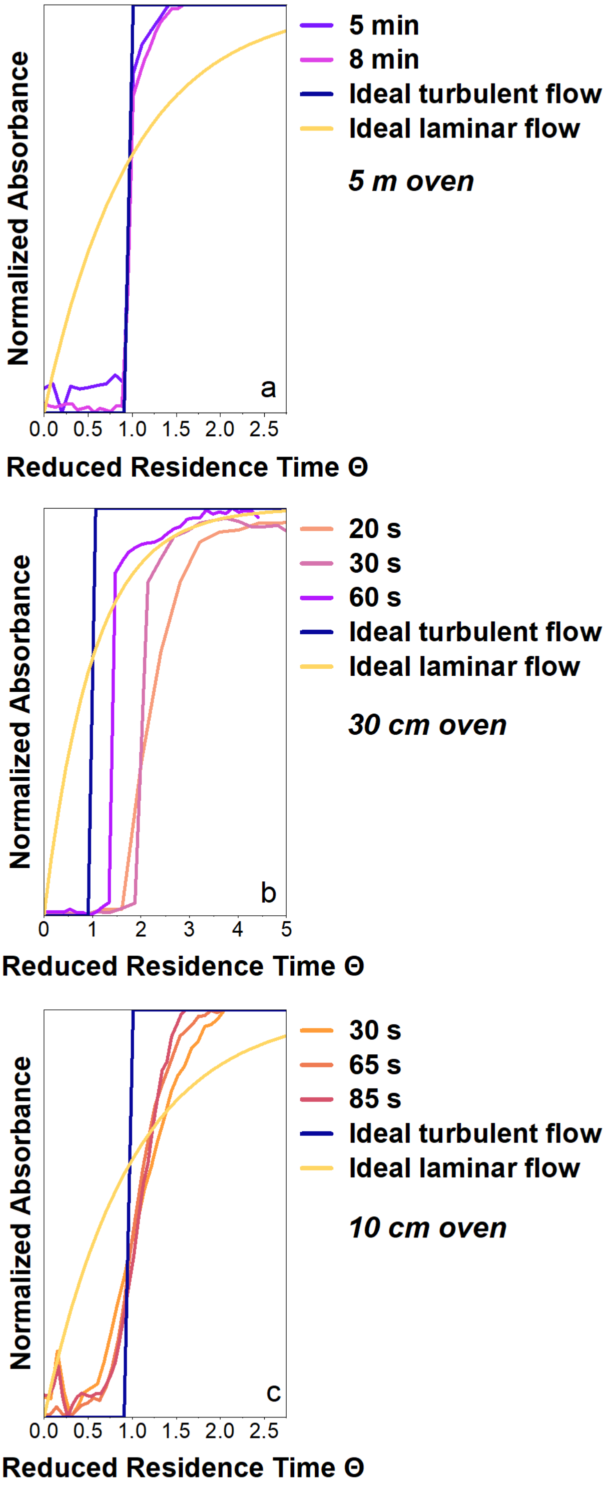

Figure S3. In situ absorbance spectra to determine the flow profile of the different reactors. (a) shows the flow profiles of the 5 m oven, (b) for the 30 cm oven, and (c) for the 10 cm oven.

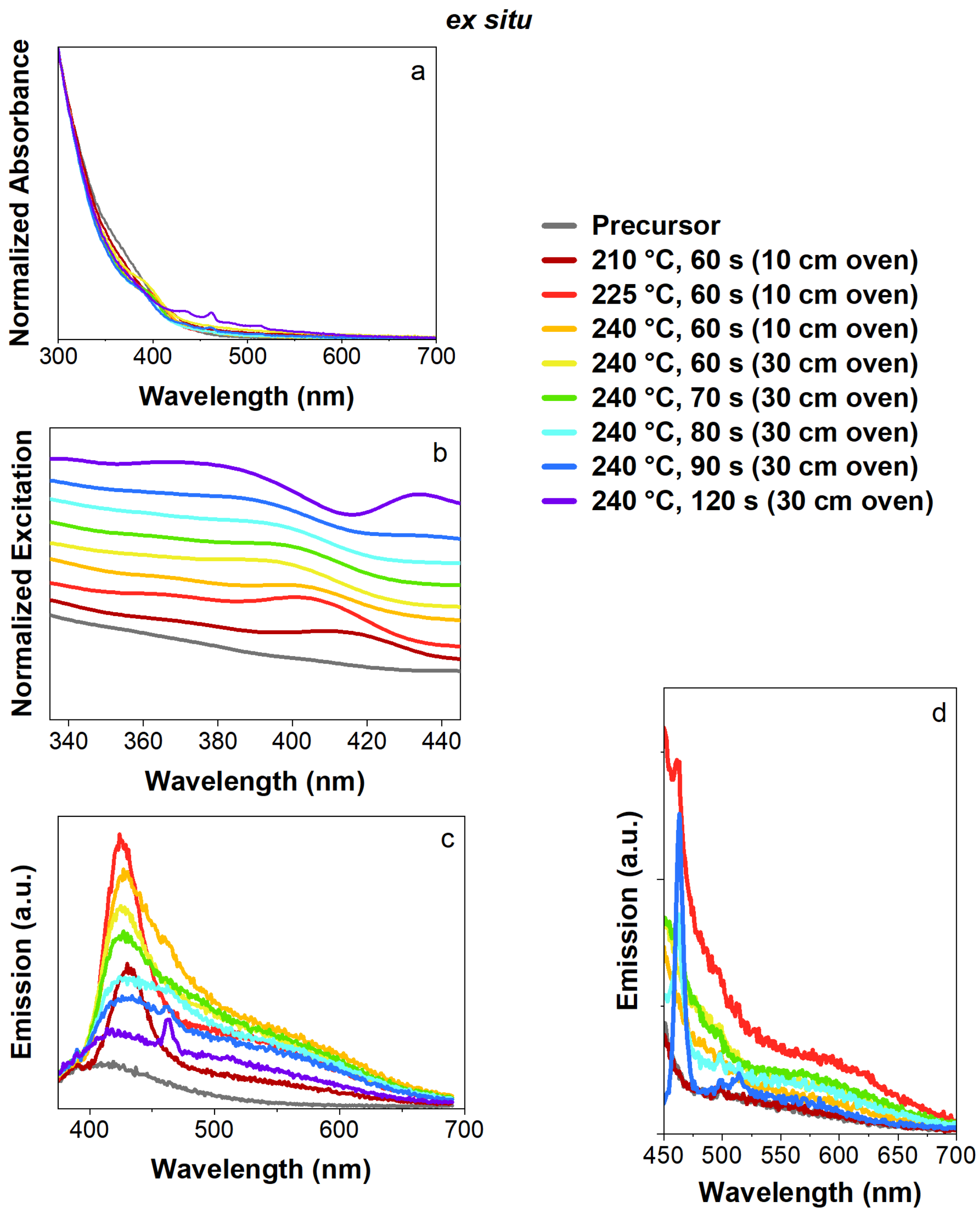

Figure S4. Ex situ measurements of flow reactor samples performed in two different oven lengths. (a) shows the normalized absorbance spectra, and (b) the excitation spectra of the flow reactor samples with an emission of 463 nm. (c) features emission spectra with an excitation wavelength of 350 nm, while (d) shows the emission spectra with an excitation wavelength of 435 nm.

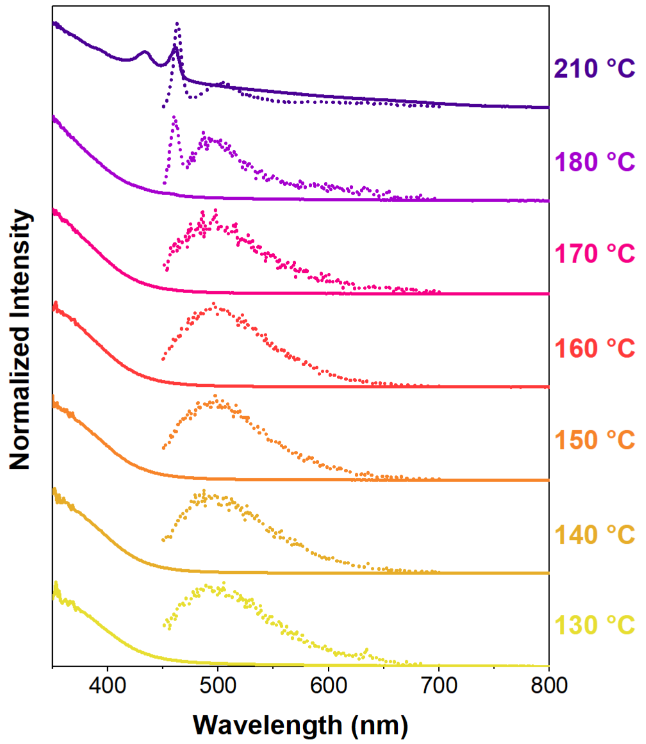

Figure S5. Ex situ absorbance and emission spectra of reactor samples synthesized in a 5 m oven with a fixed reaction time of 6 min were used to determine the nucleation temperature. The emission spectra were excited at 435 nm. Based on the results, the nucleation temperature of the CdSe NPL is 180 °C.

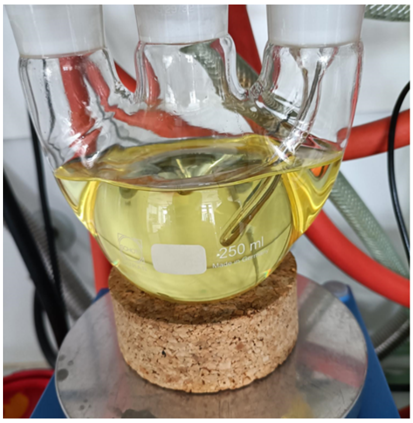

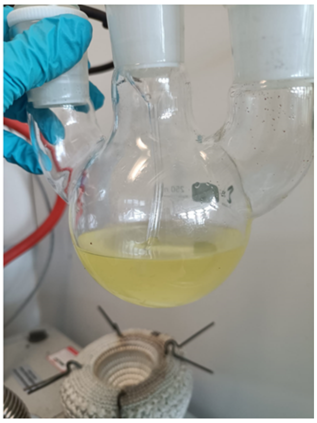

Figure S6. The reactor precursor at the beginning with AcOH added (left). After four days the reactor precursor is turning turbid (right), assumingly because cadmium acetate is forming.

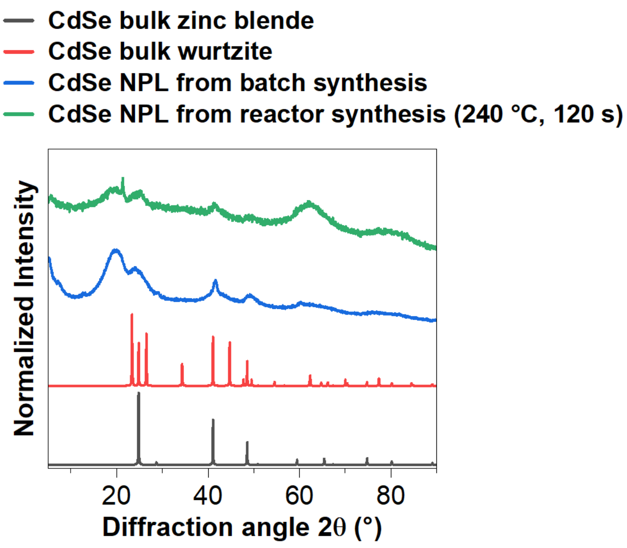

Figure S7. XRD pattern of the CdSe NPLs produced in the flow reactor and in a batch synthesis.

INFORMATION ON SAXS FITTING. The SAXS data was modeled using SasView. Here, fitting models for a standard spherical form factor,[4] power law,[5] and parallelepiped model[6,7] were used. The overall particle concentration (in vol%) and scattering contrast (between CdSe and the used solvent) were precalculated and kept constant throughout the modeling. Furthermore, for all fits, the DREAM algorithm was used. The sphere model was used for the sample at 200 °C, 20 s, and 200 °C, 60 s. A combination of the sphere and power law model was used for the following samples: 210 °C and 60 s, 220 °C and 60 s, 230 °C and 60 s, and 240 °C and 60 s. Finally, a combination of the sphere and parallelepiped model was used for the sample 240 °C and 120 s. The size distribution was varied on all samples.

REFERENCES.

(1) Zhang, F.; Ilavsky, J.; Long, G. G.; Quintana, J. P. G.; Allen, A. J.; Jemian, P. R. Glassy Carbon as an Absolute Intensity Calibration Standard for Small-Angle Scattering. *Metall Mater Trans A* **2010**, *41* (5), 1151–1158. DOI: 10.1007/s11661-009-9950-x.

(2) Pauw, B. R.; Smith, A. J.; Snow, T.; Terrill, N. J.; Thünemann, A. F. The modular small-angle X-ray scattering data correction sequence. *Journal of applied crystallography* **2017**, *50* (Pt 6), 1800–1811. DOI: 10.1107/S1600576717015096. Published Online: Nov. 29, 2017.

(3) Jain, A.; Ong, S. P.; Hautier, G.; Chen, W.; Richards, W. D.; Dacek, S.; Cholia, S.; Gunter, D.; Skinner, D.; Ceder, G.; Persson, K. A. Commentary: The Materials Project: A materials genome approach to accelerating materials innovation. *APL Materials* **2013**, *1* (1). DOI: 10.1063/1.4812323.

(4) Guinier, A.; Fournet, G.; Walker, C. B.; Vineyard, G. H. Small-Angle Scattering of X-Rays. *Physics Today* **1956**, *9* (8), 38–39. DOI: 10.1063/1.3060069.

(5) Paris, O.; Aichmayer, B.; Fratzl, P. Small-angle scattering from spherical particles on randomly oriented interfaces. *International Journal of Materials Research* **2006**, *97* (3), 290–294. DOI: 10.1515/ijmr-2006-0046.

(6) Onsager, L. THE EFFECTS OF SHAPE ON THE INTERACTION OF COLLOIDAL PARTICLES. *Annals of the New York Academy of Sciences* **1949**, *51* (4), 627–659. DOI: 10.1111/j.1749-6632.1949.tb27296.x.

(7) Nayuk, R.; Huber, K. Formfactors of Hollow and Massive Rectangular Parallelepipeds at Variable Degree of Anisometry. *Zeitschrift für Physikalische Chemie* **2012**, *226* (7-8), 837–854. DOI: 10.1524/zpch.2012.0257.
